# Serum albumin and muscle measures in a cohort of healthy young and old participants

**DOI:** 10.1007/s11357-015-9825-6

**Published:** 2015-08-27

**Authors:** E. M. Reijnierse, M. C. Trappenburg, M. J. Leter, S. Sipilä, L. Stenroth, M. V. Narici, J. Y. Hogrel, G. Butler-Browne, J. S. McPhee, M. Pääsuke, H. Gapeyeva, C. G. M. Meskers, A. B. Maier

**Affiliations:** 1 Department of Internal Medicine, Section of Gerontology and Geriatrics, VU University Medical Center, P.O. Box 7057, 1007 MB Amsterdam, The Netherlands; 2 Department of Internal Medicine, Amstelland Hospital, Amstelveen, The Netherlands; 3 Gerontology Research Centre and Department of Health Sciences, University of Jyväskylä, Jyväskylä, Finland; 4 Department of Biology of Physical Activity, University of Jyväskylä, Jyväskylä, Finland; 5 Division of Medical Sciences & Graduate Entry Medicine, MRC-ARUK Centre of Excellence for Musculoskeletal Ageing Research, University of Nottingham, Royal Derby Hospital Centre, Nottingham, The Netherlands; 6 UPMC UM 76, INSERM U 974, CNRS 7215, Institut de Myologie, Paris, France; 7 School of Healthcare Science, John Dalton Building, Manchester Metropolitan University, Manchester, M1 5GD UK; 8 Institute of Exercise Biology and Physiotherapy, Centre of Behavioural and Health Sciences, University of Tartu, Tartu, Estonia; 9 Department of Rehabilitation Medicine, VU University Medical Center, Amsterdam, The Netherlands

**Keywords:** Sarcopenia, Muscle mass, Muscle strength, Serum albumin

## Abstract

Consensus on clinically valid diagnostic criteria for sarcopenia requires a systematical assessment of the association of its candidate measures of muscle mass, muscle strength, and physical performance on one side and muscle-related clinical parameters on the other side. In this study, we systematically assessed associations between serum albumin as a muscle-related parameter and muscle measures in 172 healthy young (aged 18–30 years) and 271 old participants (aged 69–81 year) from the European MYOAGE study. Muscle measures included relative muscle mass, i.e., total- and appendicular lean mass (ALM) percentage, absolute muscle mass, i.e., ALM/height^2^ and total lean mass in kilograms, handgrip strength, and walking speed. Muscle measures were standardized and analyzed in multivariate linear regression models, stratified by age. Adjustment models included age, body composition, C-reactive protein and lifestyle factors. In young participants, serum albumin was positively associated with lean mass percentage (*p* = 0.007) and with ALM percentage (*p* = 0.001). In old participants, serum albumin was not associated with any of the muscle measures. In conclusion, the association between serum albumin and muscle measures was only found in healthy young participants and the strongest for measures of relative muscle mass.

## Introduction

The clinical relevance of sarcopenia is increasingly being recognized. Loss of muscle mass starts around the age of 30 years and has been reported to exceed losses of over 50% by the age of 80 years (Baumgartner et al. 1998; Bijlsma et al. 2012). Sarcopenia, low muscle mass is associated with functional impairment, a lower quality of life, and mortality (Landi et al. 2012; Vandewoude et al. 2012). To date, there is no consensus regarding diagnostic criteria: there is debate on which measures of muscle mass, strength, and function to include, which correction factors to apply and which cut-off points to use to determine sarcopenia in clinical practice. The use of different diagnostic criteria results in a large variety in prevalence rates of sarcopenia and differences in treatment implications (Bijlsma et al. 2013a; Janssen et al. 2002; Reijnierse et al. 2015; Van Kan 2009).

The definition of sarcopenia should be based on the strongest associated diagnostic measures with different muscle-related outcomes. Therefore, studies examining the association between diagnostic measures of sarcopenia and muscle-related outcomes are required. Serum albumin, a protein synthesized by the liver, declines with increasing age (Mühlberg and Sieber 2004; Bourdel-Marchasson et al. 2010) and it serves as a muscle-related parameter by different mechanisms. Muscle acts as a storage pool for serum albumin: it is stored extracellularly in muscles and leaks into the muscle cell immediately after exercise (McNeil and Khakee 1992; Schiaffino and Partridge 2008). Several causal mechanisms are postulated by which serum albumin influences muscle mass, i.e., by its antioxidant capacities (Oettl and Stauber 2007). Oxidative damage plays a role in the decline of muscle mass at older age by regulating both muscle protein synthesis and degradation (Derbré et al. 2014; Thompson 2009). Moreover, serum albumin is a nonspecific carrier for numerous hormones, including androgens which action affects not only muscle mass and function but also an increase in satellite cell number and myonuclei in the muscle (Sinha-Hikim et al. 2003). Studies investigating the association between serum albumin and muscle measures are controversial and have reported inconsistent results, which might be explained by the use of different muscle measures, study designs, demographic characteristics, and/or different adjustment models (Baumgartner et al. 1996; Kwon et al. 2007; Schalk et al. 2005; Schalk et al. 2004; Snyder et al. 2012; Visser et al. 2005).

In order to create evidence-based consensus on clinically valid measures of sarcopenia, associations between its candidate measures of muscle mass, strength, and function on one side and muscle-related clinical parameters on the other need to be systematically explored (Bijlsma et al. 2014; Bijlsma et al. 2013b, c, d). We hypothesized that there is an association between serum albumin as a muscle-related parameter and different muscle measures. As the muscle-related role of serum albumin is complex and prone to confounders such as comorbidity hampering correct interpretation, we aimed to study the aforementioned relation in a healthy European cohort of young and older people.

## Methods

### Study design

Data were derived from the MYOAGE study, a cross-sectional European multicenter study that included healthy young (aged 18–30 years) and healthy older (aged 69–81 years) men and women. Young and old participants were initially included to study age-related differences in muscle quality, contractile characteristics, and neural control (McPhee et al. 2013). Participants were recruited in the Netherlands (Leiden), Finland (Jyvaskyla), France (Paris), Estonia (Tartu), and the UK (Manchester) and were recruited via advertisement in newspapers, at universities, and at associations of emeriti to make sure that they were cognitively active (McPhee et al. 2013). Analyses were performed on 443 participants who had data available on serum albumin levels.

The MYOAGE study aimed to include healthy participants only to minimize the confounding effects of disease on sarcopenia. In short, exclusion criteria included a dependent living status, the inability to walk a distance of 250 m, morbidity (neurologic disorders, metabolic diseases, rheumatic diseases, recent malignancy, heart failure, severe obstructive pulmonary disease (COPD), coagulation disorders), immobilization for 1 week during the last 3 months, the use of specific medication (insulin, immunosuppressive drugs), and orthopedic surgery during the last 2 years or still causing pain or functional limitation. Those who participated in athletic events were also excluded (McPhee et al. 2013).

Local medical ethical committees of the participating medical centers approved the study. After written informed consent was obtained and participants were medically screened, measurements were performed at local study centers according to unified and standardized operation procedures (McPhee et al. 2013).

### Participant characteristics

A questionnaire was used to assess lifestyle factors such as physical activity, smoking habits, alcohol use, and education level. Education level was defined as high (university), moderate (high school), and low (primary school). Physically active was defined as exercising at least three times per week for at least 30 min. Standing height and body mass were measured for each participant. Diseases were categorized into cardiovascular disease (including cardiovascular events, hypertension, and arterial surgery), non-insulin-dependent diabetes mellitus, mild COPD, thyroid disease, and osteoarthritis. Subsequently, the sum score of diseases was determined. Cognitive function was measured using the mini-mental state examination (MMSE) (Folstein et al. 1975), and depressive symptoms were measured using the geriatric depression scale (GDS) (Yesavage and Sheikh 1986). For inclusion in the MYOAGE study, all participants needed to have a MMSE score higher than 23 points and a GDS score lower than 5 points. The use of medication was registered, and the sum score of all oral and inhaled medication was indicative of disease severity (McPhee et al. 2013).

### Serum albumin and inflammation status

Blood samples were collected in the morning, while participants were in a fasting state, and stored at −80 °C (McPhee et al. 2013). Serum albumin levels (g/L) were measured by use of a colorimetric assay using a bromocresol green assay with a Roche COBAS Integra 800 automated analyzer (Roche Diagnostics Corp., Indianapolis, USA). Calibration was performed using the traceability method, standardized against the CRM 470 reference preparation. Two-point calibration was performed after reagent lot change and following quality control procedures. Limit of detection of serum albumin was 10 to 70 g/L, lower detection limit of measurement was 2 g/L. Inter- and intra-assay coefficients of variation (CV) were 0.4 and 1.7 %, respectively. C-reactive protein (CRP) was used as a marker of inflammation and measured using an immunoturbidimetric assay (Roche COBAS Integra 800 automated analyzer, Indianapolis, USA).

### Muscle measures

#### Muscle mass

Muscle mass was determined by dual-energy X-ray absorptiometry (DXA) (Netherlands, Hologic QDR 4500, version 12.4, Hologic, Inc., Bedford, USA; Estonia, Lunar Prodigy Advanced, version EnCore 10.51.006 (GE Healthcare, UK); Finland, Lunar Prodigy, version EnCore 9.30; France, Lunar Prodigy, version EnCore 12.30; UK, Lunar Prodigy Advance, version EnCore 10.50.086). Due to these differences in equipment, data was normalized using country-specific *z* scores. Measurements were performed while participants were in a fasted state, having not eaten anything for 12 h but having had a glass of water before attending the laboratory. Alcohol intake and sauna use were not allowed for at least 24 h, and heavy or strenuous exercise was avoided for 48 h prior to the visit. Scans were performed while participants were lying in a supine position, with the arms and legs fully extended. Standard regions were drawn and manually adapted only when necessary (McPhee et al. 2013).

Measures of relative muscle mass were calculated as lean mass divided by body mass (Janssen et al. 2002) and appendicular lean mass (ALM) as the sum of lean mass of both arms and legs divided by body mass (Estrada et al. 2007). These measures were expressed in percentages. Measures of absolute muscle mass encompassed adjusted absolute muscle mass by ALM divided by height squared (ALM/height^2^) (Baumgartner et al. 1998) and total lean mass in kilograms.

#### Muscle strength

Handgrip strength was used to estimate muscle strength and measured with the Jamar Handgrip Dynamometer (Sammons Preston, Inc., Bolingbrook, IL, USA), after adjusting the width of the dynamometer to the size of the individual hand. Participants were instructed to stand upright with the handheld dynamometer, stretching the arm parallel to, but not pressed against the body. Three measurements were performed for both hands separately at a maximal performance, and the highest score was used for the analyses. Smoking was not allowed in the 2 h prior to muscle function measurements (McPhee et al. 2013).

#### Physical performance

Physical performance was measured by the 6-min walking test wherein participants were instructed to walk around cones. The cones were placed 20 m apart, with exception of France where the cones were placed 25 m apart. Participants were instructed to walk as fast as possible in Finland, Estonia, the United Kingdom (UK) and France, while they were instructed to walk at their usual pace in the Netherlands (McPhee et al. 2013). Walking speed was determined from the measured distance over a total of 6 min and expressed in meters per second (m/s).

### Statistical analyses

Statistical analyses were performed separately for young and old participants. Descriptive analyses were performed to determine participant characteristics. Variables with a Gaussian distribution are presented as mean and standard deviation (SD), while those with a non-Gaussian distribution are presented as median and interquartile range (IQR). Muscle measures were standardized by country and gender specific *z* scores to minimize the effects due to the use of different equipment and to allow comparison of effect sizes of serum albumin and their association with muscle measures.

Multivariate linear regression analyses were performed to assess the association between serum albumin and muscle measures, while taking different adjustment models stratified by age into account. In model 1, analyses were adjusted for age to exclude residual confounding by age. In model 2, analyses were further adjusted for body mass, fat mass, or height. Lean mass and ALM percentages were adjusted for body mass because a higher body mass is associated with a lower relative muscle mass (Delmonico et al. 2007; Lebrun et al. 2006). Total lean mass in kilograms and ALM/height^2^ were adjusted for fat mass because these two measures do not take fat mass into account. Handgrip strength was adjusted for body mass and height because taller people and people with a higher body mass may have a higher handgrip strength (Bassey and Harries 1993; Ramlagan et al. 2014). Walking speed was adjusted for height because taller people may have a higher walking speed (Bendall et al. 1989; Troosters et al. 1999). In model 3, analyses were further adjusted for CRP as a marker of inflammation and lifestyle factors including physically active, current alcohol use, and current smoking.

Results of the regression analyses can be interpreted as follows: a 1-g/L higher serum albumin level is associated with a β × SD score of the muscle measure. The Statistical Package for the Social Sciences (SPSS) version 17.0 was used for the analyses (SPSS Inc., Chicago, IL, USA), and *p* values of <0.05 were considered statistically significant.

## Results

A total of 172 young (mean age 23.4 years, SD 2.8) and 271 old (mean age 74.5 years, SD 3.3) participants were included. Table 1 shows the participant characteristics, stratified by age. Young and old participants had low numbers of comorbidities and medication use. No significant differences in gender and CRP levels <3 mg/L distribution were found between young and old participants. Mean serum albumin levels were 48.4 g/L (SD 3.1) in young participants and 44.8 g/L (SD 2.8) in old participants.

**Table 1:** Participant characteristics, stratified by age (*n* = 443)

	Young (*n* = 172)	Old (*n* = 271)
Age, years	23.4 (2.8)	74.5 (3.3)
Male, *n* (%)	82 (47.7)	131 (48.3)
Highly educated, *n* (%)^a^	131 (76.2)	94 (34.7)
Lifestyle factors		
Current alcohol use, *n* (%)	141 (82.0)	191 (70.7)
Current smoking, *n* (%)	24 (14.0)	13 (4.8)
Physically active, *n* (%)^b^	93 (66.4)	120 (52.4)
Comorbidity		
Nr. of diseases, median [IQR]	0 [0–0]	1 [0–1]
Nr. of medications, median [IQR]	0 [0–1]	1 [0–3]
Mental state		
MMSE score (points), median [IQR]	30 [29–30]	29 [28–30]
GDS score (points), median [IQR]	0 [0–1]	1 [0–2]
Blood parameters		
Serum albumin (g/L)	48.4 (3.1)	44.8 (2.8)
CRP <3 (mg/L), *n* (%)	150 (87.2)	232 (85.6)
Anthropometry		
Height (m)	1.73 (0.09)	1.67 (0.09)
Body mass (kg)	68.6 (12.1)	71.4 (12.7)
Fat mass (kg)	16.0 (6.8)	21.5 (7.4)
Muscle measures		
Lean mass percentage (%)^c^	73.2 (9.0)	67.3 (8.3)
ALM percentage (%)^d^	33.2 (4.7)	28.8 (4.1)
ALM/height^2^ (kg/m^2^)	7.5 (1.3)	7.2 (1.1)
Total lean mass (kg)	50.2 (11.4)	47.8 (9.9)
Handgrip strength (kg)	42.3 (12.3)	32.9 (9.4)
Walking speed (m/s)	1.85 (0.3)	1.46 (0.2)

Variables are presented as mean (SD) unless indicated otherwise

*IQR* interquartile range, *SD* standard deviation, *MMSE* mini-mental state examination, *GDS* geriatric depression scale, *CRP* C-reactive protein, *ALM* appendicular lean mass

^a^University level

^b^Exercising at least three times per week for 30 min

^c^Total lean mass as percentage of total body mass

^d^ALM as percentage of total body mass

Table 2 shows the association between serum albumin and standardized muscle measures. In young participants, serum albumin was positively associated with lean mass percentage after adjusting for body mass. This association did not change after further adjustment for CRP and lifestyle factors. Serum albumin was positively associated with ALM percentage after adjustment for age. This association strengthened after further adjustment for body mass, CRP, and lifestyle factors. Further adjustment for CRP and lifestyle factors did not change the results. Serum albumin was not associated with ALM/height^2^, handgrip strength, and walking speed in all three models. In old participants, serum albumin was not associated with any of the muscle measures.

**Table 2:** Association between serum albumin and standardized muscle measures, stratified by age (*n* = 443)

	Young (*n* = 172)			Old (*n* = 271)		
	β	SE	*p* value	β	SE	*p* value
Z Lean mass percentage (%)						
Model 1: age	0.04	0.02	0.06	−0.01	0.02	0.76
Model 2: as 1+ body mass	0.06	0.02	**0.008**	−0.01	0.02	0.64
Model 3: as 2+ CRP + lifestyle	0.07	0.02	**0.007**	−0.01	0.02	0.53
Z ALM percentage (%)						
Model 1: age	0.05	0.02	**0.017**	0.00	0.02	0.89
Model 2: as 1+ body mass	0.06	0.02	**0.002**	0.00	0.02	0.92
Model 3: as 2+ CRP+ lifestyle	0.07	0.02	**0.001**	0.00	0.02	0.80
Z ALM/height^2^ (kg/m^2^)						
Model 1: age	−0.01	0.03	0.86	0.02	0.02	0.43
Model 2: as 1+ fat mass	0.02	0.03	0.53	0.01	0.02	0.57
Model 3: as 2+ CRP+ lifestyle	0.03	0.03	0.34	0.01	0.02	0.52
Z Total lean mass (kg)						
Model 1: age	0.00	0.03	0.94	0.01	0.02	0.77
Model 2: as 1+ fat mass	0.02	0.03	0.44	0.00	0.02	0.95
Model 3: as 2+ CRP+ lifestyle	0.05	0.03	0.13	−0.01	0.02	0.57
Z Handgrip strength (kg)						
Model 1: age	0.01	0.02	0.68	0.00	0.02	0.98
Model 2: as 1+ body mass + height	0.03	0.02	0.26	0.00	0.02	0.83
Model 3: as 2+ CRP+ lifestyle	0.02	0.03	0.36	0.00	0.02	0.83
Z Walking speed (m/s)						
Model 1: age	0.01	0.02	0.77	−0.02	0.02	0.29
Model 2: as 1+ height	0.00	0.02	0.85	−0.02	0.02	0.39
Model 3: as 2+ CRP+ lifestyle	0.02	0.02	0.42	0.00	0.02	0.97

All muscle measures were standardized into country and gender specific *z* scores. All *p* values were assessed with linear regression analysis and adjusted in separate models. Lifestyle factors included physically active, current alcohol use, and current smoking. Bold entries are significant values of *p* < 0.05. Interpretation: a 1-g/L higher serum albumin level is associated with a β × SD score of the muscle measure; e.g., a 1-g/L higher serum albumin level is associated with 0.36 (0.04 bèta *z* score × 9.0 SD) higher lean mass percentage. SD scores of muscle measures: lean mass percentage: young 9.0%, old 8.3%; ALM percentage: young 4.7%, old 4.1%; ALM/height^2^: young 1.3 kg/m^2^, old 1.1 kg/m^2^; total lean mass: young 11.4 kg, old 9.9 kg; handgrip strength: young 12.3 kg, old 9.4 kg; walking speed: young 0.3 m/s, old 0.2 m/s

*β* bèta, *SE* standard error, *CRP* C-reactive protein, *ALM* appendicular lean mass

## Discussion

The aim of the present study was to investigate the association between serum albumin and different muscle measures. In young participants, serum albumin was positively associated with both measures of relative muscle mass after adjustments for age, body mass, CRP, and lifestyle factors. Serum albumin was not associated with both measures of absolute muscle mass, handgrip strength, and walking speed in young participants. In old participants, serum albumin was not associated with any of the muscle measures.

In the present study, serum albumin was not associated with measures of absolute muscle mass and handgrip strength in young and old participants. Results from previous studies are controversial. In old participants, a positive association was found between serum albumin and relative and absolute muscle mass (Baumgartner et al. 1996; Visser et al. 2005). In accordance with our findings, a cross-sectional and a longitudinal study also failed to find an association between serum albumin and muscle strength in community-dwelling older participants (Kwon et al. 2007; Snyder et al. 2012). However, a longitudinal study has found a negative association between low baseline serum albumin and a decline in handgrip strength in community-dwelling old participants (Schalk et al. 2005). Aforementioned studies included large heterogeneous populations with various physical activity levels and health status in contrast to our healthy population. In the present study, we did not find an association between serum albumin and walking speed. In line with this result, previous studies also failed to find an association between serum albumin and walking speed (Schalk et al. 2004; Singh et al. 2014).

The present population of healthy old participants was the result of a rigorous selection. Only healthy and fit older persons were included, thus eliminating the effects of morbidity as much as possible and preventing other factors than muscle aging alone to interfere with the association between serum albumin and muscle measures. The result of this selection may be reflected by the low standard deviations of both serum albumin and muscle measures in the old participants, which were even lower than in the young participants. It could be that the resulting limited contrast contributed to the absence of associations between serum albumin and muscle measures in older group. However, in contrast to our results, previous studies did report an association between serum albumin and relative and absolute muscle mass in old participants, while the studies also reported low standard deviations of serum albumin levels (Baumgartner et al. 1996; Visser et al. 2005). A negative association was found between a lower baseline serum albumin (mean 40.0 g/L, 3.1 SD) and a decline in ALM percentage in community-dwelling people aged 70 years and older (Visser et al. 2005). Therefore, results of the former studies might be driven by other factors than muscle aging itself. Literature does not support our expectations that in relevant geriatric populations, such as community-dwelling older adults (mean 43.1 g/L, 2.7 SD) (Takata et al. 2012) and nursing home residents (mean 40.1 g/L, 4.4 SD) (Onem et al. 2010); the variation in serum albumin is higher thereby inducing associations with muscle measures. However, these studies did not address the association between serum albumin and muscle measures.

There are several mechanisms, which may interfere with the association between serum albumin and muscle measures which can be hypothesized to exist in both directions. A positive association of relative muscle mass in young participants indicates the importance of body composition in the relation between serum albumin and muscle. In old participants, both serum albumin and muscle measures were lower compared to young participants. Direction of causality could change with advanced age. For example, change of body composition may turn a dominant role of the reservoir function of muscle tissue for albumin in younger subjects into a less prominent role in older subjects in which relative muscle mass is lower. This is in concordance with the association of relative muscle mass in younger subjects. Mechanisms in the opposite direction can also be hypothesized. Low serum albumin could lead to more antioxidant capacities (Oettl and Stauber 2007), causing more oxidative damage and therefore more muscle breakdown (Derbré et al. 2014; Thompson 2009). Another mechanism could be the activation of the phosphatidyl-inositol 3-kinase pathway by serum albumin (Jones et al. 2003), leading to muscle hypertrophy (Lai et al. 2004) and therefore an increase in muscle mass. Serum albumin may affect serum testosterone (Vermeulen et al. 1996) and low free testosterone was found to be associated with low absolute muscle mass, expressed as ALM/height^2^ (Yuki et al. 2013). However, total testosterone was not associated with low absolute muscle mass in an earlier study (Yuki et al. 2013). Due to the cross-sectional design of the study, causal relations cannot be inferred. Therefore, we refrained from extensive exploration of this topic. As we cannot demonstrate these causal mechanisms due to the cross-sectional design, we assume the change in body composition could explain the differences between the young and old participants.

Several strengths and limitations of the present study should be addressed. Since the MYOAGE study consisted of healthy participants, the influence of diseases on muscle measures was minimized. Furthermore, previous studies only investigated the association between serum albumin and muscle strength (Kwon et al. 2007; Schalk et al. 2005; Snyder et al. 2012) or muscle mass (Baumgartner et al. 1996; Visser et al. 2005). The present study examined these associations simultaneously and additionally included physical performance. In addition, we adjusted for CRP as proxy for inflammation, which is in contrast to other studies (Kwon et al. 2007; Schalk et al. 2004; Snyder et al. 2012; Starling et al. 1999). It is important to take inflammation into account because it is associated both with a decline in muscle strength (Schaap et al. 2009) and muscle mass (Malafarina et al. 2012). Our study was limited by the use of a cross-sectional study design whereby causality cannot be proven. The cohort consisted of a relatively small but very defined sample size. Serum albumin was measured by use of a colorimetric assay instead of nephelometry as the golden standard. However, colorimetric assays are often used in daily clinical practice (Infusino and Panteghini 2013).

In conclusion, serum albumin as a muscle-related parameter was positively associated with both measures of relative muscle mass in healthy young participants, while serum albumin was not associated with any of the muscle measures in healthy old participants. Future studies should systematically investigate the association of other muscle-related clinical parameters with muscle measures to create evidence-based consensus of sarcopenia.
